# From “Serum Sickness” to “Xenosialitis”: Past, Present, and Future Significance of the Non-human Sialic Acid Neu5Gc

**DOI:** 10.3389/fimmu.2019.00807

**Published:** 2019-04-17

**Authors:** Chirag Dhar, Aniruddha Sasmal, Ajit Varki

**Affiliations:** ^1^Departments of Medicine and Cellular and Molecular Medicine, University of California, San Diego, La Jolla, CA, United States; ^2^Glycobiology Research and Training Center, University of California, San Diego, La Jolla, CA, United States

**Keywords:** Neu5Gc, anti-Neu5Gc, serum sickness, xenosialitis, red meat, sialic acid, inflammation, antibodies

## Abstract

The description of “serum sickness” more than a century ago in humans transfused with animal sera eventually led to identification of a class of human antibodies directed against glycans terminating in the common mammalian sialic acid *N-*Glycolylneuraminic acid (Neu5Gc), hereafter called “Neu5Gc-glycans.” The detection of such glycans in malignant and fetal human tissues initially raised the possibility that it was an oncofetal antigen. However, “serum sickness” antibodies were also noted in various human disease states. These findings spurred further research on Neu5Gc, and the discovery that it is not synthesized in the human body due to a human-lineage specific genetic mutation in the enzyme *CMAH*. However, with more sensitive techniques Neu5Gc-glycans were detected in smaller quantities on certain human cell types, particularly epithelia and endothelia. The likely explanation is metabolic incorporation of Neu5Gc from dietary sources, especially red meat of mammalian origin. This incorporated Neu5Gc on glycans appears to be the first example of a “xeno-autoantigen,” against which varying levels of “xeno-autoantibodies” are present in all humans. The resulting chronic inflammation or “xenosialitis” may have important implications in human health and disease, especially in conditions known to be aggravated by consumption of red meat. In this review, we will cover the early history of the discovery of “serum sickness” antibodies, the subsequent recognition that they were partly directed against Neu5Gc-glycans, the discovery of the genetic defect eliminating Neu5Gc production in humans, and the later recognition that this was not an oncofetal antigen but the first example of a “xeno-autoantigen.” Further, we will present comments about implications for disease risks associated with red meat consumption such as cancer and atherosclerosis. We will also mention the potential utility of these anti-Neu5Gc-glycan antibodies in cancer immunotherapy and provide some suggestions and perspectives for the future. Other reviews in this special issue cover many other aspects of this unusual pathological process, for which there appears to be no other described precedent.

## First Reports of “Serum Sickness” in Humans Infused With Animal Serum

Following the discovery of the effectiveness of tetanus and diphtheria antitoxins by Emil von Behring and Shibasaburo Kitasato, the popularity of serotherapy soared in the 1880s and 1890s (1). However, reports of reactions to the diphtheria antitoxin also started to appear. In 1899, Bolton reported 100 cases of reactions to the diphtheria antitoxin (2). Pirquet and Schick suggested the use of the phrase “serum sickness” in their book *Die Serumkrankheit* (3) recognizing that the reactions were against animal serum components present in the antitoxin preparations.

## “Serum Sickness” Patients Have “H-D” Antibodies, Some of Which Recognize Neu5Gc-Containing Glycans Found in Human Cancers

### The Initial Definition of “H-D” Antibodies

Two decades later, Hanganutziu and Deicher independently described human antibodies that agglutinated animal erythrocytes (4, 5). These Hanganutziu-Deicher antibodies (H-D antibodies) were prominent in subjects with serum sickness who had received therapeutic animal antisera. Subsequently, similar antibodies were reported in patients with no prior exposure to animal sera but instead suffering from other diseases (6).

### A Portion of H-D Antibodies Are Directed Against Neu5Gc-Containing Glycans, but HD Antigens Can Also Be Present in Diseased Human Tissues

About 50 years later, two groups independently showed that a portion of these heterophile H-D antibodies recognized gangliosides containing the sialic acid *N*-Glycolylneuraminic acid (Neu5Gc) (7, 8). This sialic acid was later shown to be derived from the common mammalian sialic acid *N*-Acetylneuraminic acid (Neu5Ac) by the addition of a single oxygen atom that is added to CMP-Neu5Ac in a complex cytosolic reaction catalyzed by the enzyme cytidine monophosphate *N*-acetylneuraminic acid hydroxylase (*Cmah*) (9–13). The definition of H-D antibodies sparked further research and these were then detected in the sera of patients with multiple pathological conditions, including rheumatoid arthritis, infectious mononucleosis, leprosy, syphilis, leukemia, Kawasaki disease (a disease that causes inflamed blood vessels), and various cancers (14–24).

### Generation of H-D Antibodies in Chickens, Confirming H-D Antigens in Human Cancers

Early on, it was also noted that anti-H-D serum of high titer could be generated in chickens immunized with H-D antigen-active glycosphingolipid, *N*-Glycolylneuraminyl-lactosylceramide (purified from equine erythrocytes) (18, 25). Immunohistochemistry or thin-layer chromatography using these polyclonal antibodies as well as indirect methods such as inhibition of bovine erythrocyte agglutination by human H-D antiserum were then used to confirm the presence of Neu5Gc-glycans in meconium and multiple human tumors (14–24). Paradoxically, the H-D antigens or Neu5Gc-glycans were also found on human tissue gangliosides and glycoproteins (18, 25–35). Much later, work from our group resulted in further affinity purification of such chicken polyclonal antibodies (36) (during the process we have noted that the bovine serum albumin preparation originally used as a “carrier” for the immunogen is contaminated with bovine serum glycoproteins bearing Neu5Gc-glycans, which also contribute importantly to the immune response in chickens). These preparations were used as a valuable tool for the detection of smaller amounts of Neu5Gc-glycans present even in normal human tissues (36, 37), particularly on epithelia lining hollow organs (the origin of carcinomas), and on endothelia (where atherosclerotic cardiovascular disease occurs).

## Humans Cannot Synthesize Neu5Gc

### Humans Are Genetically Deficient in *CMAH*, the Primary Enzyme That Generates Neu5Gc

**Figure 1:**
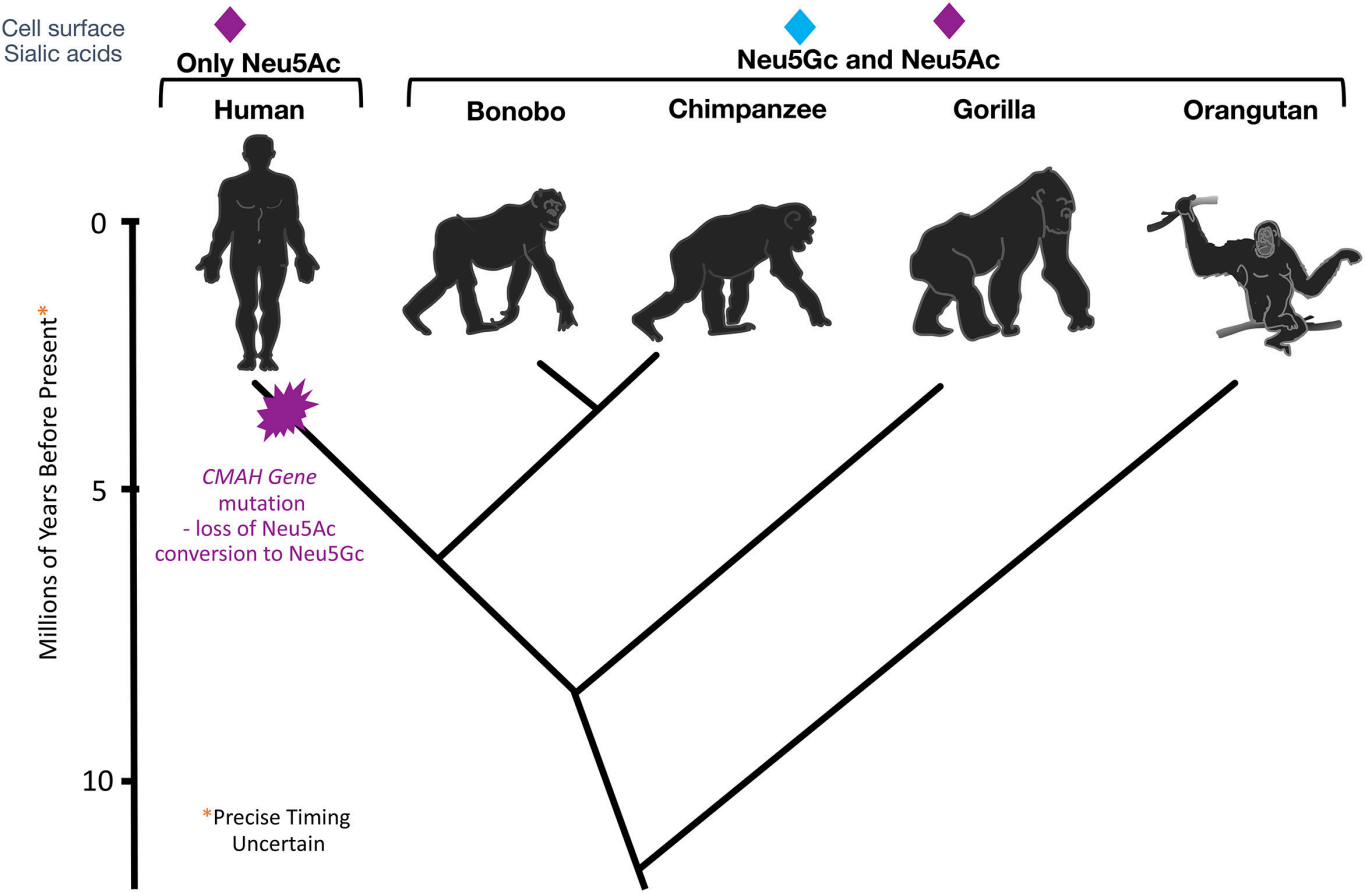
Evolutionary Loss of *CMAH*. Multiple methods of analysis indicate that the *CMAH* mutation occurred about 2–3 mya after the divergence from the Pan group.

These findings inspired further work on *CMAH*, and the discovery of an inactivating mutation that likely got fixed in the human lineage >2 million years ago. All humans were found to be homozygous for a deletion of exon 6 in the *CMAH* gene (38, 39) and this deletion was later shown to have been mediated by a single Alu-Alu fusion event (40). While the first published report incorrectly claimed that the mutation resulted in an altered reading frame and a large non-functional fusion protein (38), the second report the same year (41) showed that it actually results in a greatly truncated form of the enzyme. Comparisons with our closest living evolutionary relatives (42) indicated that this mutation occurred after our common ancestry with these “great apes” (Figure 1).

### Possible Selection Mechanisms for the Initial Hominin Mutation in *CMAH*

**Figure 2:**
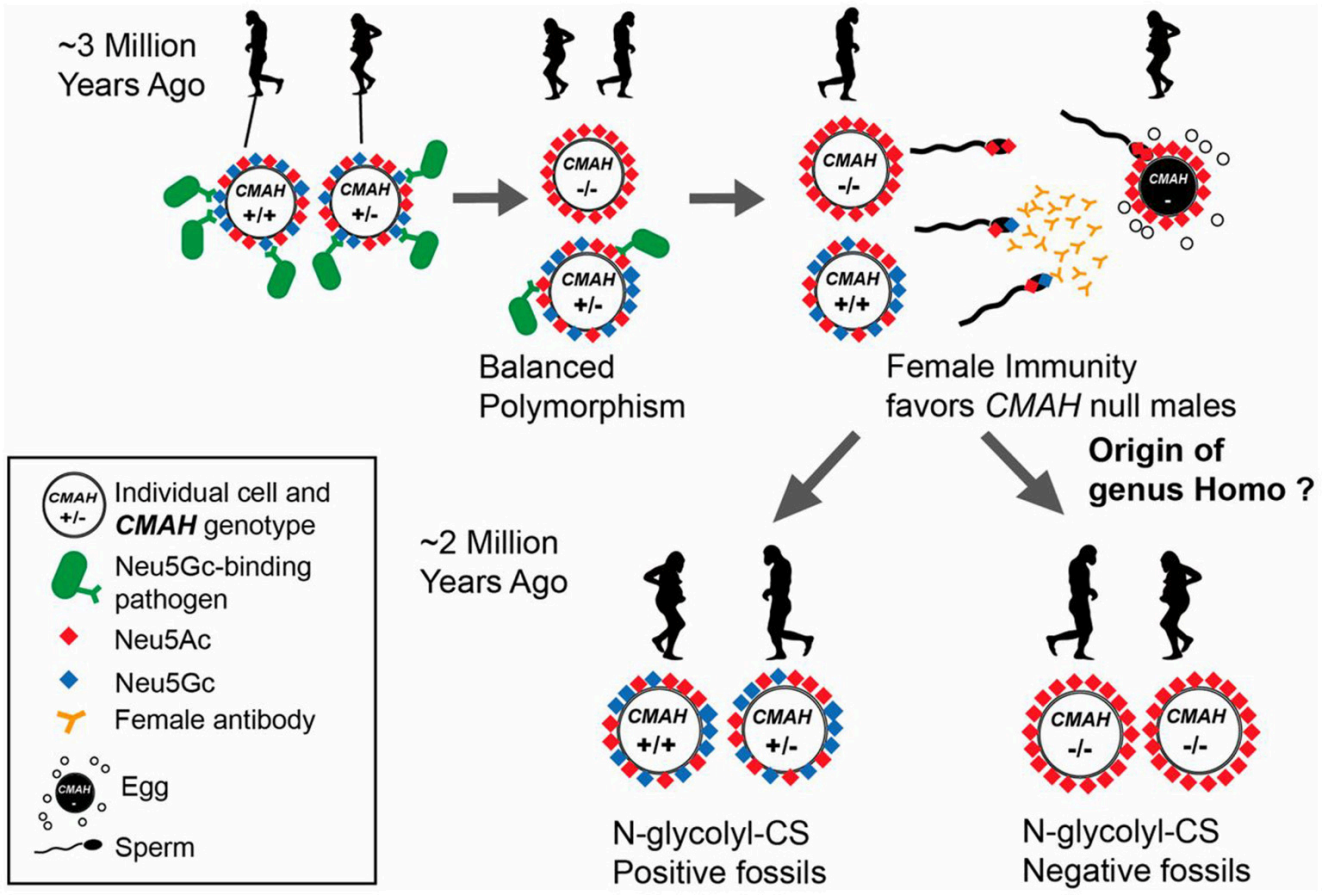
Potential scenario for the role of Neu5Gc loss and female anti-Neu5Gc immunity in the origin of the genus *Homo* via interplay of natural and sexual selection acting on cell-surface Sias. There are many known pathogens that recognize and exploit Neu5Gc (blue diamond) as a receptor on host target cells. Natural selection by such pathogens may have selected for rare *CMAH* null alleles that abolish Neu5Gc expression in homozygote individuals. Such individuals have only Neu5Ac and its derivatives on their cells (red diamonds) allowing an escape from pathogens, but at higher frequencies would be targeted by adapting pathogens, resulting in maintenance of a balanced polymorphism. *CMAH*^−/−^ females with anti-Neu5Gc antibodies also present in their reproductive tract would favor sperm from *CMAH*^−/−^ males due to anti-Neu5Gc antibody-mediated cryptic selection against *CMAH*^+/−^ or *CMAH*^+/+^ males expressing Neu5Gc on their sperm. Once the frequency of the *CMAH* null allele reaches a critical level, this process can drive fixation of the loss-of-function allele in a population by directional selection. Figure and figure legend reproduced from Bergfeld et al. (45).

Whether this mutation got fixed in the human lineage as a result of positive or negative selection is still a matter of speculation. A pandemic caused by a lethal infectious pathogen that preferred to bind to Neu5Gc leading to negative selection is one possible explanation (43). Another mutually non-exclusive possibility is selective fertility of Neu5Gc-deficient females with Neu5Gc-deficient males, leading to positive selection of this genotype (44). This so-called “cryptic female choice” theory (44) is pictorially depicted in Figure 2 (The figure legend details this theory) (45).

This mechanism was demonstrated in human-like *Cmah* null mice (44, 46). On the other hand, a random *CMAH* mutation may simply have become fixed in a small group of individuals who eventually gave rise to modern humans. Regardless, this inactivation of *CMAH* lead to drastic changes in the sialoglycome that likely pre-dated the origin of the genus *Homo* (44). Given that Neu5Gc has been found in multiple species of the deuterostome lineage ranging from sea urchins to non-human primates, *CMAH* is at least 500 million years old (47). Interestingly, Neu5Gc was independently lost in multiple lineages including sauropsids (birds and reptiles), monotremes (platypus) and certain other lineages (47, 48). More details about the evolutionary implications of Neu5Gc and anti-Neu5Gc glycan antibodies have been covered by P. Gagneux in another review in this special issue.

## Humans Express Dietary-Derived Neu5Gc on Their Cell Surfaces

### Neu5Gc-Glycans Are Present in Smaller Amounts in Normal Human Epithelia and Endothelia

**Figure 3:**
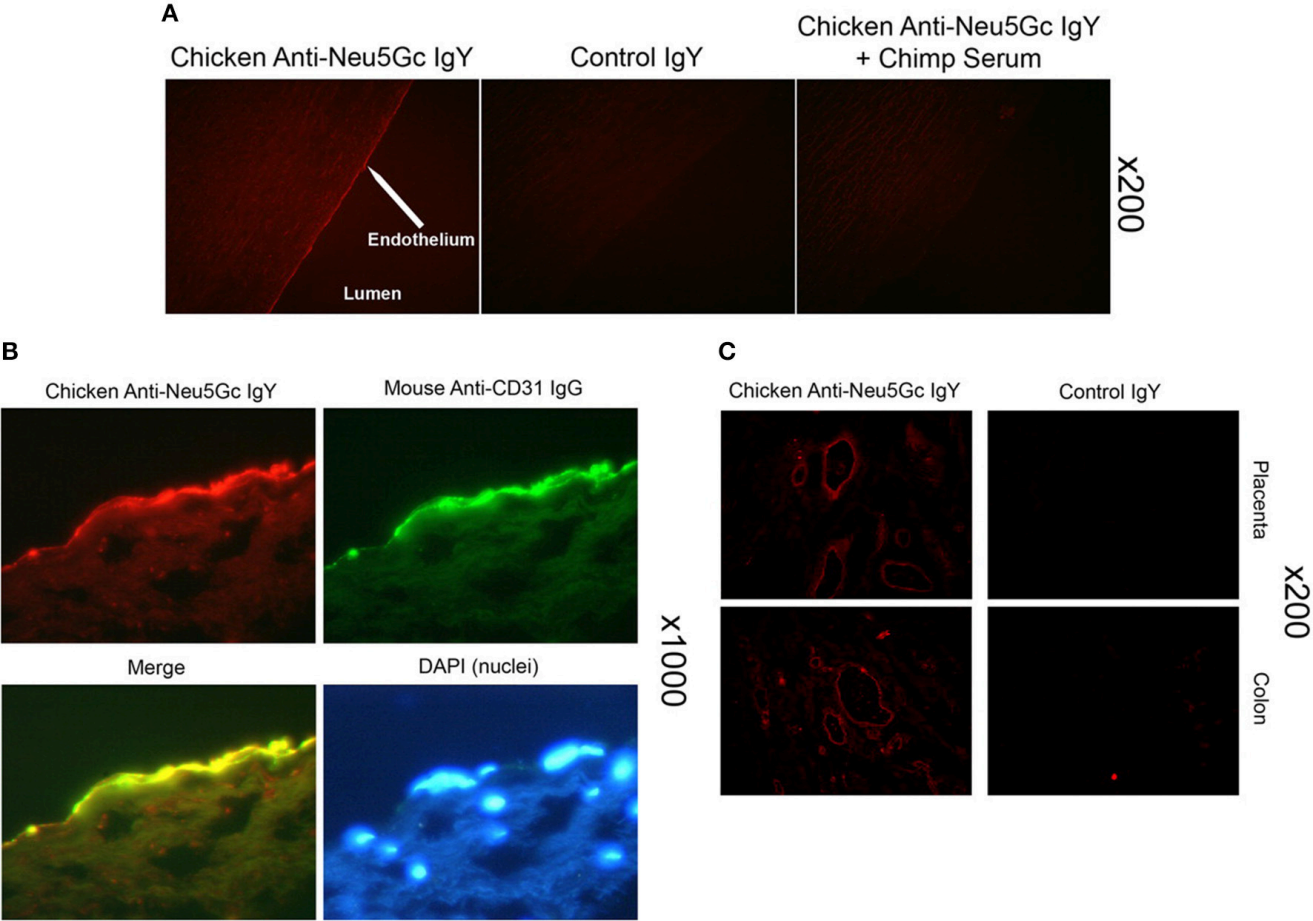
Detection of Neu5Gc in aortic endothelium of human autopsy samples and microvasculature of colon and placenta. The chicken anti-Neu5Gc antibody (cGcAb) was used to detect the presence of Neu5Gc on the endothelium of autopsy samples of normal-appearing human aorta. Typical representatives of 8 autopsy samples studied are shown. The red Cy3 fluorescence represents labeling of endothelial cells of the aorta. **(A)** Specificity of the antibody was demonstrated by the lack of signal with the non-immunized control chicken IgY (middle) and the abrogation of signal by adsorption with Neu5Gc-rich glycoproteins of chimpanzee serum (right). Magnification ×200. **(B)** Sections were double-stained with anti-CD31 for endothelial cells and counterstained with DAPI to visualize nuclei (magnification ×1000). **(C)** Sections of placenta (top) and colon (bottom) stain for Neu5Gc along microvasculature endothelial lining with the use of cGcAb. Control IgY (right) demonstrates specificity of signal (magnification ×200). Figure and figure legend reproduced from Pham et al. (37).

Apart from onco-fetal human tissue, very small amounts of Neu5Gc-glycans were surprisingly also found to be incorporated in normal human secretory epithelia and small and large vessel endothelia (36, 37, 49) (Figure 3). Concurrent mass-spectrometric studies of purified sialic acids confirmed the presence of Neu5Gc (49) and in *N-*glycans released from tumor samples (50).

### Neu5Gc-Glycans in *CMAH* Null Humans and Mice Are Exclusively Derived From Food Sources

Although human cells cultured in FCS have been reported to express Neu5Gc-glycans (42, 51) this appears to be due to metabolic incorporation or passive adsorption of glycoconjugates. So far it seems that the only source of exogenous Neu5Gc in human and humanized *Cmah* null mice is via dietary intake (49, 50, 52, 53) Sialic acids have never been detected in plants and are found in large amounts primarily in vertebrates and a few “higher” invertebrates as well as in some insects (54–58). The occurrence of Neu5Gc in poultry and fish is rare but common in some milk products and greatly enriched in red meats (49, 53, 59, 60).

### Red Meat as the Primary Dietary Source of Neu5Gc–The First Example of a “Xenoautoantigen”

With no other explanation for the presence of Neu5Gc-glycans in human tissues as confirmed in the mouse model, it was concluded that humans incorporate Neu5Gc from dietary sources. Studies using a DMB-HPLC assay to detect Neu5Gc showed its enrichment in beef, pork and lamb (53). Additionally, all humans produce anti-Neu5Gc glycan antibodies in varying titers (61). In light of these antibodies that likely bind to any incorporated Neu5Gc-glycans, this is the first example of a “xenoautoantigen.” This state, with both the presence of Neu5Gc-glycans as well as the corresponding anti-Neu5Gc glycan antibodies has been called “Xenosialitis” and likely plays a role in multiple human pathologies, as elaborated in later sections of this review.

### Mechanisms of Neu5Gc Uptake and Incorporation Into Human Tissues and Cells

When human volunteers ingested free Neu5Gc, it was shown to be largely excreted in the urine (49). Extended feeding of *Cmah* null mice with free Neu5Gc in drinking water also did not result in efficient tissue incorporation except in a malignant tumor (52). In contrast, feeding of glycosidically-bound Neu5Gc attached to porcine mucins gave low-level incorporation into normal tissues over a period of weeks (62). While it has been previously shown that *N*-glycolylmannosamine a degradation product of Neu5Gc which may more easily be taken up than the parental sialic acid (63), the exact mechanism by which bound Neu5Gc from the diet results in metabolic incorporation is not known and requires further investigation.

**Figure 4:**
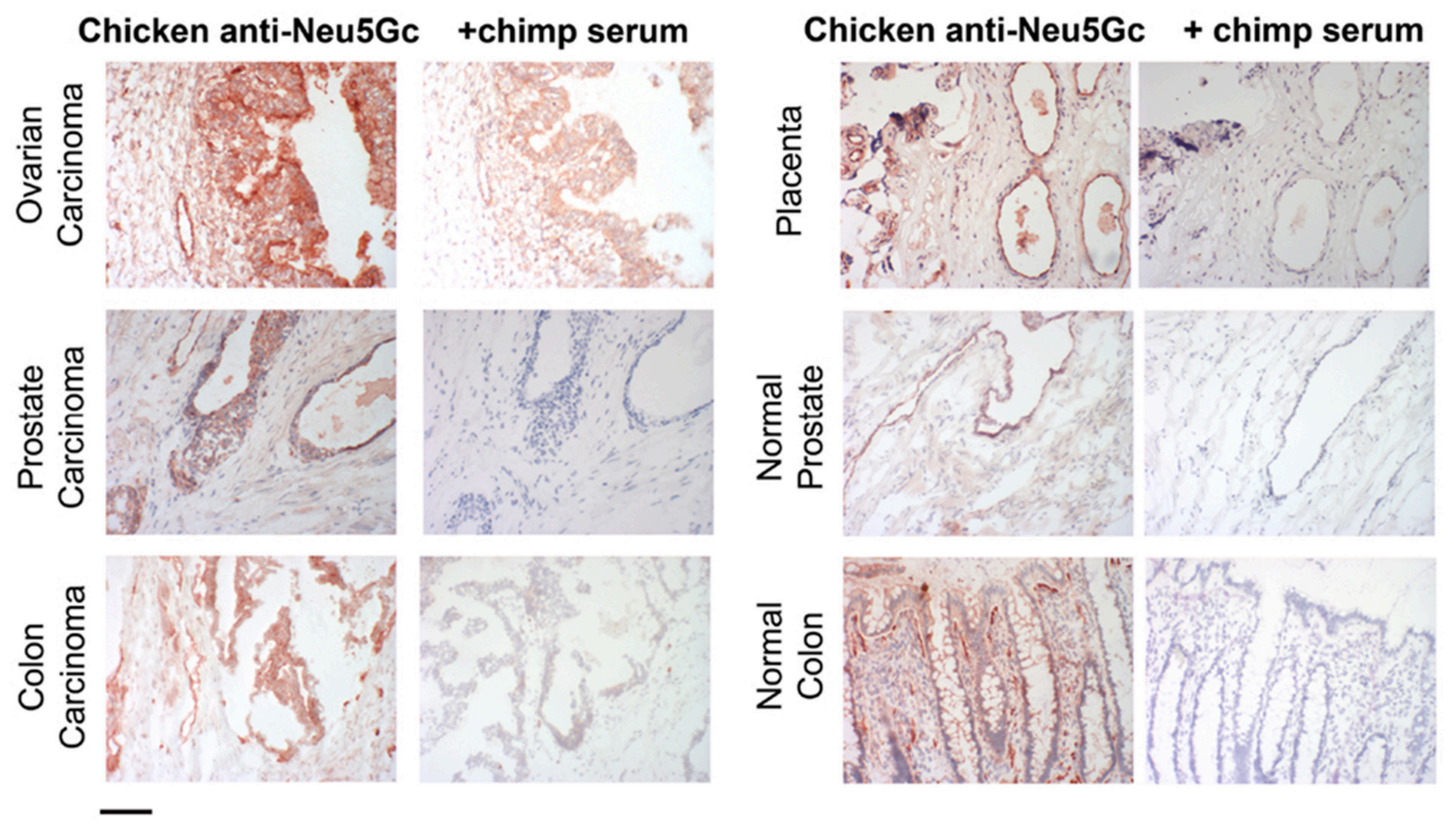
Examples of incorporation of Neu5Gc in malignant and healthy human tissue. Expression of Neu5Gc is observed to be enhanced in malignant epithelia as seen here in carcinomas of the ovary, prostate and colon (left panel). In contrast, expression of Neu5Gc in normal tissue is seen in the ducts of the prostate gland and in the epithelial lining of the colon (Right panel). Endothelial cells of the normal placenta are used here as a positive control for Neu5Gc immunostaining. As a negative control, the binding is blocked competitively with Neu5Gc-containing chimpanzee serum. Magnification used was 200× and scale bar is 100 μm. Figure and figure legend reproduced from Samraj et al. (65).

In contrast, human epithelial cells in culture can metabolically incorporate free or bound Neu5Gc and express it into endogenous glycoproteins (64) (Figure 4). The mechanism of uptake and incorporation of the Neu5Gc into human epithelial cells (derived from a primary colon carcinoma), fibroblast, and neuroblastoma cells was shown to be dependent on non-clathrin-mediated pinocytic pathways (64). Free Neu5Gc taken up by pinocytosis, or bound Neu5Gc released by a lysosomal sialidase, can then be exported to the cytosol by the lysosomal sialic acid transporter. Activation of the resulting cytosolic free Neu5Gc by the CMP-sialic acid synthase then generates the donor for incorporation into glycoconjugates in the Golgi apparatus, on newly synthesized glcoconjugates. The reason why free Neu5Gc gives incorporation in cultured cells but not in the intact organism is because of the rapid clearance by the kidney in the latter situation. The difference between free and bound Neu5Gc is also relevant to recognition by antibodies which can only interact with the latter. Moreover, the typical antibody binding site can accommodate glycan chains of 4–6 monosacharride (66). Antibodies typically cannot efficiently recognize just a terminal Neu5Gc even when glycosidically bound. For this reason, many studies that have utilized simple alpha-linked Neu5Gc as a target in ELISA assays grossly underestimate the amount and complexity of anti-Neu5Gc glycan antibody response (67). Hereafter, we therefore refer to antibodies against glycosidically-bound Neu5Gc as “anti-Neu5Gc-glycan antibodies” which are diverse and complex because of the underlying glycans.

### Metabolic Fate of Neu5Gc

As the reaction catalyzed by Cmah is irreversible, all mammalian cells must have pathways to adjust cellular Neu5Gc levels to their needs to avoid continued accumulation. We discovered a metabolic pathway for the turnover of exogenous Neu5Gc in human cells (68). It was shown that cytosolic extracts harbor the enzymatic machinery to sequentially convert Neu5Gc into *N*-glycolylmannosamine, *N*-glycolylglucosamine, and *N*-glycolylglucosamine 6-phosphate, whereupon irreversible de-*N*-glycolylation of the latter results in the ubiquitous metabolites glycolate, and glucosamine 6-phosphate. Later, it was shown that metabolic turnover of the dietary Neu5Gc in humans and *Cmah* null mice modifies chondroitin sulfate and this stable *N-*Glycolyl chondroitin sulfate (Gc-CS) survives even in ancient fossils (45). This discovery opened a door for “ancient glycomics” and could help in tracking early human lineages and their food habits. Additionally, we are working on developing a simplified assay to measure levels of Gc-CS in serum to predict red meat-related incorporation.

Parallel studies of the *P. falciparum* malarial protein VAR2CSA that mediates parasite attachment to the placental trophoblast led to discovery of the target “oncofetal chondroitin sulfate” (ofCS) which is not detected in normal tissues, but is shared by many types of cancers and can be detected using recombinant VAR2CSA(rVAR2) (69–72). As this pattern is similar to that of Neu5Gc-glycans in placental and tumor tissue, it was natural to suspect that it might be related to Gc-CS. However, this matter requires further investigation.

## Humans Also Have Anti-Neu5Gc Antibodies

### All Humans Have Circulating Anti-Neu5Gc-Glycan Antibodies

All human adults have varying levels of circulating IgM, IgG, and IgA antibodies against Neu5Gc-glycans (49, 61, 73–75). Human anti-Neu5Gc glycan antibodies interact with metabolically incorporated Neu5Gc to promote chronic inflammation, likely contributing to tumor inflammation and cancer progression (50, 53) and vascular inflammation (37).

### Origin of Human Anti-Neu5Gc-Glycan Antibodies

Our group later showed that human anti-Neu5Gc glycan antibodies appear during the first year of life and correlate with the introduction of Neu5Gc in the diet (76). Sera from infants aged 0–12 months were analyzed, and anti-Neu5Gc IgM and IgG antibodies against Neu5Gcα2-6Lac started to appear at the time these infants were weaned on to cow's milk-based formula. Interestingly, anti-Neu5Gc IgM antibodies were absent at birth and at 3 months, appeared at 6 months and the levels stabilized at 12 months. There was no difference in anti-Neu5Gc IgM and IgG titers between male and female subjects. The absence of anti-Neu5Gc IgM antibodies in cord blood sera suggests that anti-Neu5Gc antibodies are not germ-line encoded “natural” antibodies (77) that occur naturally in human and other mammals, but instead require a postnatal antigenic stimulus. Anti-Neu5Gc antibodies are likely to be affinity matured antibodies as has been shown earlier (78). However, spontaneous generation of anti-Neu5Gc IgM or IgG antibodies in *Cmah* null mice did not occur even when large quantities of Neu5Gc were fed to them. This is despite the presence of relatively hyper-reactive B cells, apparently caused by the loss of Neu5Gc-containing Siglec ligands (79, 80). On the other hand, deliberate immunization with an artificial immunogen rich in Neu5Gc, such as chimpanzee RBCs, and complete Freund's adjuvant, did elicit anti-Neu5Gc IgM, and IgG antibodies in *Cmah* null, but not in wild type mice (50, 75).

### *N*-Glycolyl Groups Are Rare in Nature, Increasing the Likelihood of Antigenicity

*N*-acetyl groups are common in nature (PubMed search of “*N*-Acetyl” gives >30,000 citations), often originating from the donor acetyl-CoA. In contrast, a search of “*N*-Glycolyl” gives ~270 citations, which are either about Neu5Gc or about *N*-Glycolylmuramic acid, found in certain bacterial peptidoglycans (81–86). The *CMAH* gene is a distant homolog of prokaryotic genes generating UDP-*N*-glycolylmuramic for peptidoglycan biosynthesis (82, 83). In both instances, a mono-oxygenase reaction is involved. It is unclear why glycolyl-CoA formed during fatty acid beta-oxidation (87, 88) is never utilized to make *N*-glycolyl groups. Regardless, the rarity of this modification makes it more likely to be antigenic. *N*-glycolylmuramic acid occurs in Freund's adjuvant (which has mycobacterial products), which we use to immunize *Cmah* null mice against Neu5Gc-glycans, but we do not observe anti-Neu5Gc Abs in mice given only adjuvant.

### Markedly Different Antigenicity of Glycosidically-Bound vs. Free Neu5Gc and Impact of Underlying Glycans

As was touched upon earlier, the difference between free and bound Neu5Gc is also relevant to Ab recognition, which can only interact with the latter. Moreover, since the typical Ab binding site accommodates 4 to 6 monosaccharides (66, 89, 90), Neu5Gc-dependent Abs cannot efficiently recognize a terminal glycosidically-bound Neu5Gc by itself. Thus, studies that utilized simple alpha-linked Neu5Gc as a target in assays (67, 91–95) grossly underestimate the complexity of the human anti-Neu5Gc Abs, which are diverse and complex, because of variations in underlying glycans (61, 96, 97). Recently, it has also been shown that the presentation mode of Neu5Gc-containing glycans in various assays affects recognition by anti-Neu5Gc glycan IgGs (98).

### Possible Mechanism of “Xenoauto-Immunization” by Microbes Like *Haemophilus influenzae*

While humans develop antibodies against Neu5Gc-containing glycans during infancy, the mechanism of immunization is still unclear. One possible explanation is “xeno-autoimmunization” by microbes such as *H. influenzae*, that normally colonize humans. Non-typeable *H. influenzae* (NTHi) like all other known microbes cannot synthesize Neu5Gc but has been shown to be able to incorporate trace amounts of free sialic acids into its cell-wall LPS (99). Also, anti-Neu5Gc antibodies appear in infants around the same time as antibodies against NTHi (76). One likely source of Neu5Gc for these microbes is foods of mammalian origin used for weaning. Indeed, NTHi was shown to be able to incorporate Neu5Gc from baby foods (76).

### A Parallel but Inconsistent Literature About Anti-tumor MAbs Against (Neu5Gc)GM3

An extensive literature originating primarily from one group (100–113) claims that a Neu5Gc-version of ganglioside GM3 (Siaα2-3Galβ1-4Glcβ1-1'-Ceramide) is tumor-specific, and cancer vaccines and MAbs (idiotypic and anti-idiotypic) targeted against it are even in clinical trials (114, 115). Until recently this group assumed that expression was unrelated to dietary intake, and that the antigen is absent from normal cells. Moreover, a collaborating group recently suggested that hypoxia induces *de novo* synthesis of (Neu5Gc)GM3 in human cells through a poorly defined “*CMAH* domain substitute” (116). However, hypoxia also increases uptake and incorporation of Neu5Gc, and fetal calf serum contains Neu5Gc. Once human cancer cells are placed in Neu5Gc-free human serum, for several passages, we find that all traces of Neu5Gc disappear. Moreover, our broad-spectrum polyclonal monospecific chicken anti-Neu5Gc Ab cannot detect any Neu5Gc in *Cmah* null mice on a Neu5Gc-free diet. Further confusion arises because the original group also uses these antibodies to treat tumors in *Cmah* wild type mice (107), which already have a large amount of endogenously synthesized Neu5Gc-GM3. This is also true of preclinical toxicity studies done in *CMAH*-positive monkeys (117). We may be misunderstanding something about this body of work, but our present assumption is that the tumor-associated (Neu5Gc)GM3 being targeted arises from dietary Neu5Gc. Alternatively, the actual epitope may be different. Regardless of the final resolution, it does not change the basic underlying hypothesis driving our current work, on red meat-derived Neu5Gc-induced “xenosialitis.”

## Anti-Neu5Gc Antibodies in Disease States

**Figure 5:**
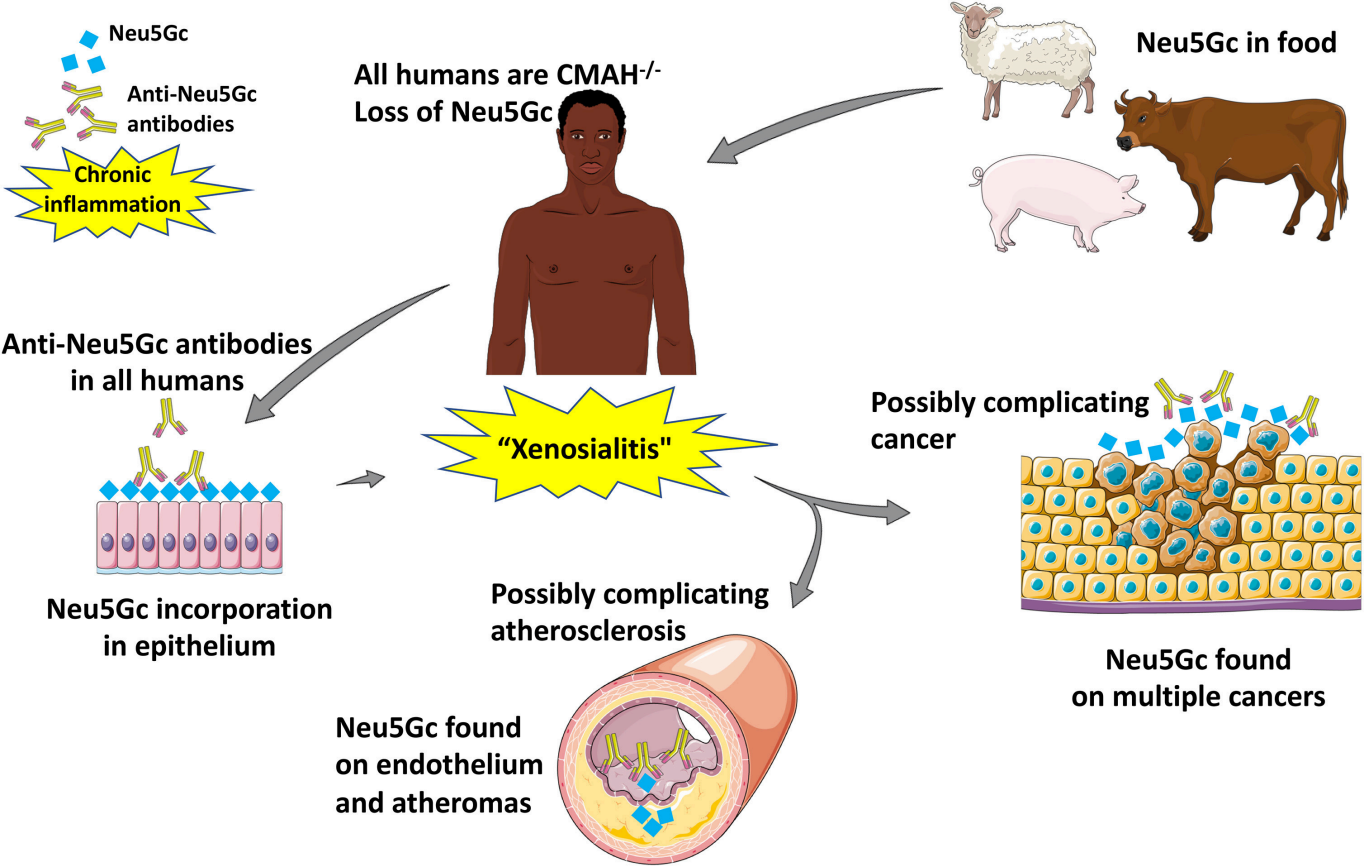
Suggested pathological implications of Neu5Gc consumption, accumulation, and subsequent inflammation in atherosclerosis and cancer [Image created with objects sourced from Servier Medical Art (http://smart.servier.com/), licensed under a Creative Common Attribution 3.0 Generic License].

As alluded to earlier, anti-Neu5Gc antibodies have been described in a multitude of diseases. Anti-Neu5Gc antibodies have broad implications in transplantation (93, 118–125) which will be covered in a separate review in this special issue. While transplantation can be associated with high levels of anti-Neu5Gc-glycan antibodies due to ATG serum therapy and/or the xenotransplant itself, these are very unusual clinical states with associated immunosuppression and other pathologies. Also of note, the phenomenon of “hormesis” has been documented with these antibodies, with very highly levels having the opposite effects e.g., killing of tumors (126, 127). In this review, we will focus on a possible role of moderate levels of the antibodies in two diseases that otherwise normal humans are particularly prone to develop: epithelial cancers (carcinomas) and atherosclerosis (Figure 5).

### Carcinomas

Accumulation of Neu5Gc-glycans has been detected in human tumors such as breast, colon, ovary, and prostate carcinomas (49, 65, 128, 129). Distinctly, red meat is enriched with bound forms of the Neu5Gc. Numerous epidemiological studies concluded that consumption of red meat is associated with atherosclerotic cardiovascular diseases and an increased risk of cancer (130, 131). Recent findings involving the Health Professionals Follow-up Study and the Nurses' Health Study cohorts confirmed that a higher intake of red meat (specifically processed red meat products) was associated with a significantly elevated risk of cancer, prominently colorectal cancer (132). The epidemiological data ruled out alternate factors such as (a) high-fat intake (133); (b) the production of heterocyclic amines and polycyclic aromatic hydrocarbons (134); (c) the presence of mutagenic *N*-nitroso compounds (135), that were once believed to be the major promoter of carcinogenesis. Our laboratory has shown that the Neu5Gc and anti-Neu5Gc-glycan antibody interaction induced “xenosialitis” may promote chronic inflammation leading to cancer progression (53).

Another possibly related carcinogenic mechanism arising from red meat was revealed by the isolation of a number of small DNAs obviously derived from specific plasmids of Acinetobacter bacteria from commercially available cow milk samples by de Villiers and zur Hausen (136–138). These authors suggest that such infections with autonomously replicating plasmids early in life are risk factors for human colon and breast cancers several decades later (139), that incorporated Neu5Gc from dietary sources might provide receptors for the viruses, and that antibodies against these viral proteins may work in concert with Neu5Gc-induced “xenosialitis.”

As has been shown earlier, inflammation and associated activation of the immune system can promote carcinogenesis (inflammation-induced cancer) and cancer progression (140–142). The seminal review on the hallmarks of cancer by Hanahan and Weinberg also mentions tumor-promoting inflammation as one of the enabling factors of cancer (143). Moreover, growing tumors induce an inflammatory response that can support cancer progression (cancer-related inflammation) (140, 144). Chronic inflammation in auto-inflammatory diseases and diet-induced metabolic syndrome is also an important etiological factor for the development of cancer (142, 145). Hence it is not surprising that red meat consumption and the “Western diet” have often been associated with increased circulating markers of inflammation in human population studies (146). Cell surface glycosylation is heavily altered in cancer cells, as seen in malignant tissue that incorporate Neu5Gc (62, 64, 147). Thus, anti-Neu5Gc antibodies likely support cancer progression by enhancing tumor-related inflammation via induction of “xenosialitis” in the humanized mouse model (*Cmah*^−/−^) (53, 148, 149). A recent study showed that there is no increase in colon cancer risk following anti-Neu5Gc antibody induction with Neu5Gc-bearing rabbit anti-T cell IgG (ATG) in recipients of kidney (150). However, there was no estimation regarding red meat intake in this study and patients with renal failure are typically advised to reduce meat intake. Furthermore, some such patients are also under immunosuppression, which would alter outcomes^*^.

Sialoglycan microarray studies enabled us to differentiate between controls and patients with various carcinomas including prostate, ovary, endometrium, colon, lung, and pancreas with regard to antibodies against Neu5Gc-Sialyl-Tn (96). A recent nested case-control study from our laboratory assessed the association between total anti-Neu5Gc antibodies and the risk of colorectal cancer (CRC) in the Nurses' Health Study cohort. This study showed that the sum total of polyclonal anti-Neu5Gc glycan antibodies were associated with CRC risk (97).

### Atherosclerosis

Myocardial infarctions (MIs), ischemic heart disease, strokes and peripheral vascular disease in humans are primarily caused by atherosclerotic cardiovascular disease (CVD) (151). Chimpanzees, our closest evolutionary cousins, on the other hand suffer from “heart attacks” as a result of idiopathic interstitial myocardial fibrosis (152). Additionally, captive chimps do not get human-like MIs despite major risk factors such as dyslipidemia and hypertension (152). There is a clear association between consumption of red meats and processed meats with increased risk of CVD in humans (131, 153). While multiple theories for this association have been put forward including cholesterol and saturated fat (154), conversion of choline and carnitine into proatherogenic Trimethylamine N-oxide (TMAO) (155–157), and oxidative damage due to heme iron (158–161), these mechanisms appear not to be specific for red meats as explained in an earlier review from our laboratory (162). “Xenosialitis,” unlike these theories, is specific to red meats and may contribute to the uniquely human severity of complications of atherosclerosis. Earlier studies from our lab have shown that Neu5Gc can be detected in the endothelium overlying the atherosclerotic plaque as well as the sub-endothelium (37). Further, human endothelial cells fed with Neu5Gc and subsequently exposed to serum containing anti-Neu5Gc glycan antibodies led to IgG and complement deposition which in turn led to increased endothelial activation, increased cytokine production, and selectin expression, events associated with early atherogenesis. These effects were inhibited by Neu5Gc-alpha-methyl glycoside, a specific competitor to anti-Neu5Gc antibodies. *Cmah*^−/−^ mice also showed Neu5Gc accumulation in their endothelium when fed with Neu5Gc (62). We are currently studying *Cmah*^−/−^ mice bred into a low-density lipoprotein knockout (Ldlr^−/−^) background fed with Neu5Gc and immunized with Neu5Gc bearing antigens to see if they have a higher risk of developing atherosclerosis as compared to controls fed Neu5Ac. Large human cohort studies are also necessary to confirm the role of anti-Neu5Gc antibodies in CVD.

## Clinical Application of Anti-Neu5Gc Glycan Antibodies

### Possible Therapeutic Role of Neu5Gc-Antigens and Anti-Neu5Gc Antibodies

Despite the possible pathogenic effects of these antibodies as described above, anti-Neu5Gc antibodies may also be potentially utilized as anti-cancer immunotherapeutic agents. Tumor cells are aberrantly sialylated and the content of sialic acid on these cells goes up markedly when compared to cells of healthy tissue (163, 164). This upregulation may explain why ingested Neu5Gc preferentially accumulates in cancer tissue (49, 62). There is also an upregulation of sialyl-Tn antigen (165–169), an epitope not commonly found (165, 170, 171) or “hidden” by *O-*acetylation of sialic acid (166) in healthy human tissue. Recent findings also show the presence of Sialyl-Tn in stem-like cells in cancer cell lines (172) and therapeutic benefits of antibodies that target these epitopes in patient-derived xenograft models of Ovarian carcinoma (173). If Neu5Gc-Sialyl-Tn is found to be relatively cancer specific, it may be used to image or even treat cancers. Indeed, *in vitro* assays have shown that human antibodies against Neu5Gc-Tn antigen purified from IVIG activate antibody-dependent cellular and complement-dependent cytotoxicity (ADCC and CDC) (96).

Another approach that has been tried is vaccination with (Neu5Gc)GM3 along with outer membrane protein complex of *Neisseria meningitidis* in proteoliposomes leading to antibody production in advanced stage breast cancer patients in a phase I study (174). A mouse-monoclonal antibody directed against (Neu5Gc)GM3, 14F7 was isolated (129) and further, has been humanized (175). 1E10, the corresponding anti-idiotype to 14F7, named racotumomab has also been tried in humans (176) and also shown to have non-apoptototic cytoxic effects *in vitro* (177). This antibody is able to bind to multiple malignant tissues including skin cancers, neuroectodermal tumors, genitourinary cancer, non-small cell lung cancer, and gastrointestinal tumors (178–182) and multiple human trials have also been conducted (e.g., NCT01598454, NCT01460472, NCT02998983, NCT01240447). However, as mentioned earlier, these studies do not make any direct link to dietary Neu5Gc, and the antibodies are reported to work even in *Cmah* wild-type mice, which have a vast excess of Neu5Gc antigens on normal tissues.

Despite all these efforts to develop effective immunotherapeutics, no efforts have been taken to control Neu5Gc consumption in cancer patients. Notably, if cancer patients are encouraged to reduce Neu5Gc consumption, a “washout” of Neu5Gc may occur in normal tissue. Following this, IV Neu5Gc may be used to “feed” tumors followed by an antibody that recognizes Neu5Gc-containing epitopes to now “find” the tumor. “Feeding” tumors is possible as Neu5Gc preferentially accumulates in malignant tissue due to increased micropinocytosis (64), rapid growth rates and hypoxic upregulation of the sialin transporter (147). This “feed-and-find” approach may turn out to be more effective than the present approaches. Additionally, monoclonal antibodies targeting Neu5Gc-containing glycans may be tested on an advanced sialoglycan microarray (183) and coupled with a newly developed computational methods (184) to confirm specificity.

Importantly, Neu5Gc has also been found in cancer therapeutic agents. Monoclonal antibodies such as trastuzumab, cetuximab and rituximab are integrated in today's cancer therapies (185). Glycosylation of these antibodies may involve Neu5Gc-rich media and/or mammalian cells that express Neu5Gc (186). Our laboratory has previously shown that incorporation of Neu5Gc in cetuximab enhanced the formation of immune complexes promoting drug clearance (187). Avoidance of Neu5Gc during production of glycoproteins may improve half-life of these antibodies while also reducing their immunogenicity.

### Biomarkers in Pathological States

Anti-Neu5Gc glycan antibodies could serve as potential biomarkers for diseases associated with red meat consumption including carcinomas, atherosclerosis, and type 2 diabetes (188–192). Current biomarkers for cancer lack sufficient sensitivity and importantly the specificity for early diagnosis (193, 194). Although antibodies against tumor-associated antigens are commonly found in cancer patients at an early stage and could potentially be sensitive detectors for malignant transformation (195, 196), none of the previously described autoantibodies show sufficient specificity in screening. Given the incorporation and display of Neu5Gc by tumor cells, the detection of Neu5Gc body-burden and antibody response together might serve as a potential biomarker for early carcinoma detection. It has been demonstrated that comparison of anti-Neu5Gc antibody levels can be used to differentiate between controls and patients with various carcinomas (96, 97). Increased anti-Neu5Gc antibody levels were also found in patients with Kawasaki disease (197).

## Conclusions and Perspectives

**Figure 6:**
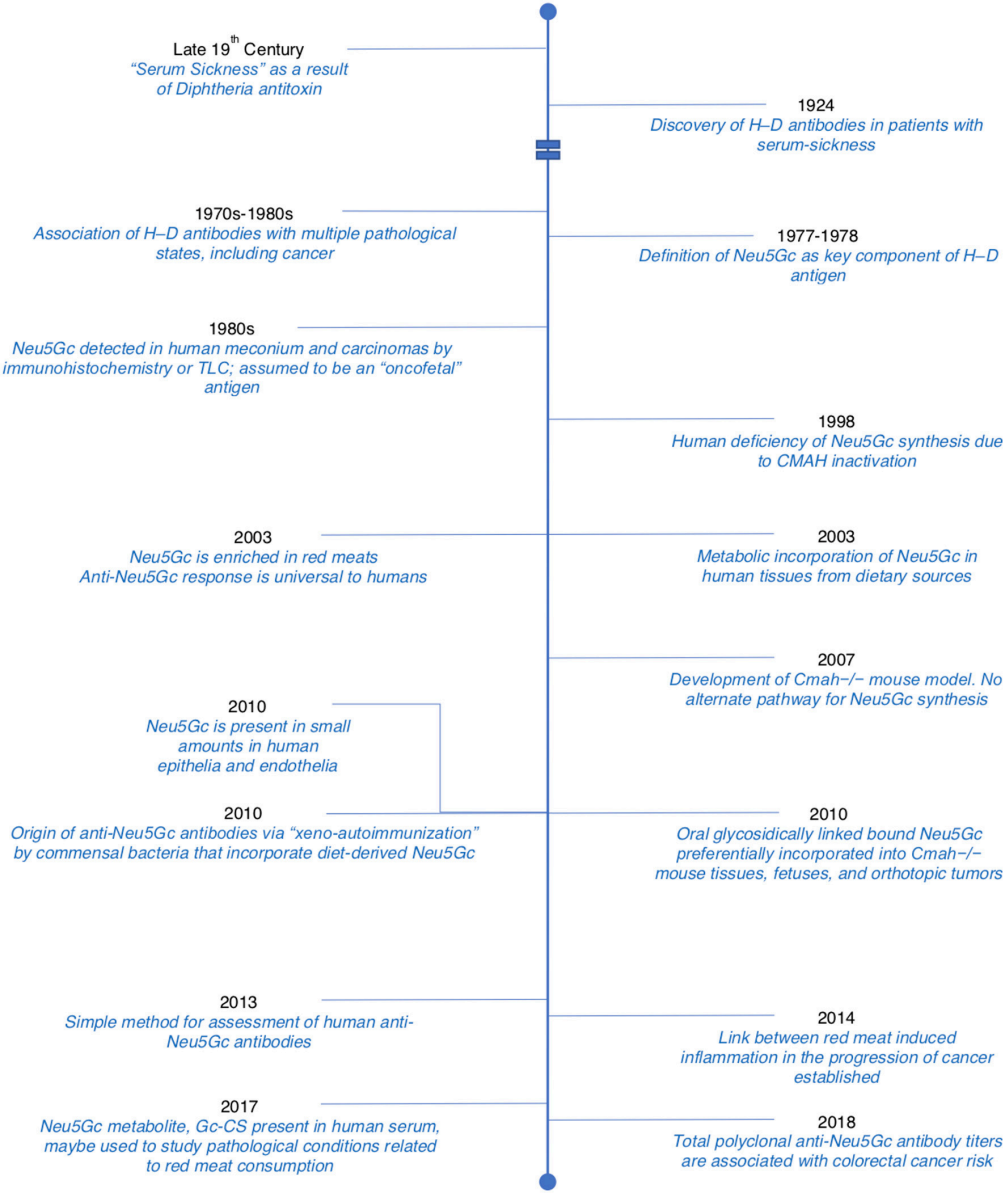
Timeline detailing important discoveries related to Neu5Gc and anti-Neu5Gc antibodies. Adopted and modified from Samraj et al. (65).

In this review, we have discussed important milestones from the early description of “Serum-sickness” as being due to antibodies directed against Neu5Gc epitopes all the way to the present-day therapeutic implications of these antibodies in cancer therapy. Some of these milestones have been represented in a concise timeline (Figure 6). While the “Xenosialitis” hypothesis is well-supported in the human-like mouse models, it has yet to be conclusively proven in humans. It remains to be seen if “Xenosialitis” plays a role in other uniquely-human diseases.

There also remain certain unresolved complexities of food sources of Neu5Gc and their propensity for metabolic incorporation. It is noteworthy that processed red meat is much more closely associated with disease risk than red meat *per se*. This is usually explained on the basis of preservatives added to process red meat. However, the same preservatives are added to other foods but are not associated with the same disease risks. One possible explanation is that the predigested nature of the processed food enhances absorption and incorporation of Neu5Gc. In this regard, there is currently no assessment of the relative impact of different foods and food processing on absorption in general. What is needed is that the equivalent of a glycemic index for the impact of glucose uptake (198, 199), i.e., “a GCemic index.” Along the same lines we are also missing an equivalent of the HbA1c (198, 199) as an index of long-term metabolic incorporation. We are currently studying the novel metabolite *N*-Glycolyl-chondroitin sulfate as a candidate.

It is also important to emphasize that there are other dietary sources of Neu5Gc besides red meat. While poultry is completely free of Neu5Gc, low levels are found in “fish” (which typically refers to the fish muscle). However, it is well-known that other food sources such as fish eggs, sea urchins, goat milk etc. can be high in Neu5Gc, and antibody development and xenosialitis in societies that consume large amounts of such foods needs to be studied further. Of course, the presence of bound Neu5Gc does not automatically equate to metabolic incorporation.

One other important perspective from these studies on Neu5Gc and anti-Neu5Gc antibodies is the consumption of red meat. With red meat being the richest source of Neu5Gc, abstaining may be the best way to prevent any “xenosialitis” induced pathologies though this would be largely improbable to sustain in the general population. Another possible way to prevent Neu5Gc uptake is to breed genetically-modified *CMAH* null livestock. Like humans, these animals will be unable to synthesize Neu5Gc and thereby prevent human dietary incorporation. But besides worries about “GMOs,” one dangerous implication of rearing such livestock is their increased susceptibility to pathogens that bind Neu5Ac which also likely affect humans. This may be combated by growing GMO modified *CMAH*^−/−^ “cultured meat” that does not synthesize Neu5Gc under strict aseptic conditions. Other alternatives include competing with an excess of the human sialic acid Neu5Ac.

## Author Contributions

All authors listed have made a substantial, direct and intellectual contribution to the work, and approved it for publication.

### Conflict of Interest Statement

The authors declare that the research was conducted in the absence of any commercial or financial relationships that could be construed as a potential conflict of interest. The handling editor declared a past co-authorship with one of the authors, AV.
